# Skeletal Muscle Mass Loss Leads to Prolonged Mechanical Ventilation and Higher Tracheotomy Rates in Critically Ill Patients

**DOI:** 10.3390/jcm13247772

**Published:** 2024-12-19

**Authors:** Gabriel M. Allgayer, Bernhard Ulm, Andreas P. Sauter, Stefan J. Schaller, Manfred Blobner, Kristina E. Fuest

**Affiliations:** 1 Department of Anaesthesiology & Intensive Care Medicine, Klinikum Rechts der Isar, School of Medicine and Health, Technical University Munich, 81675 Munich, Germany; 2 Faculty of Medicine, Department of Anesthesiology and Intensive Care Medicine, University Hospital Ulm, 89070 Ulm, Germany; 3 Department of Diagnostic and Interventional Radiology, Klinikum Rechts der Isar, School of Medicine and Health, Technical University Munich, 81675 Munich, Germany; 4 Department of Anaesthesia Intensive Care Medicine and Pain Medicine Division of General Anaesthesia and Intensive Care Medicine, Medical University of Vienna, 1090 Vienna, Austria

**Keywords:** artificial respiration, mechanical ventilation, critical illness, intensive care units, x-ray computed tomography, skeletal muscle

## Abstract

**Background**: Skeletal muscle mass depletion adversely affects critically ill patient outcomes. Standardized methods for assessing muscle mass in this population are limited, particularly regarding changes during ICU stays and their implications for risk stratification. **Methods**: In this secondary analysis of our prospective data registry of surgical ICU patients, we used a single slice extracted from a computed tomography scan to determine the patient’s direction of absolute change in skeletal muscle mass between two different time points (−14 d to +0 d and +5 d to +21 d) during his or her critical illness. **Results**: In total, 98 surgical patients were included in the final analysis. A decrease in a patient’s skeletal muscle mass is associated with prolonged mechanical ventilation compared to patients whose skeletal muscle mass remained the same or increased (415 vs. 42 h, *p* = 0.003). Patients losing skeletal muscle mass also needed to be ventilated more frequently (88.3% vs. 60.5%, *p* = 0.002), had a higher rate of tracheotomy (50.0% vs. 23.7%, *p* = 0.011), and had an increased ICU length of stay (22 vs. 13 days, *p* = 0.045). **Conclusions**: A decreased skeletal muscle index in early critical illness negatively impacts ventilation parameters, highlighting the importance of monitoring and managing muscle mass changes to optimize outcomes in ICU patients.

## 1. Introduction

Sarcopenia is referred to in the latest report of the European Working Group on Sarcopenia in Older People (EWGSOP) as a “generalized skeletal muscle disease that is associated with an increased likelihood of negative consequences such as falls, fractures, physical disability and mortality” [1]. Traditionally, sarcopenia is classified as primary, or age-related, or secondary when precipitating factors such as acute illness or injury are present [1].

While sarcopenia has traditionally been considered a consequence of chronic disease and aging [1], recent studies show that it plays a critical role in the outcomes of patients in the ICU. Mortality in elderly ICU patients [2], in-hospital and 6-month mortality in ICU patients [3], as well as hospital mortality in mechanically ventilated critically ill patients are increased by sarcopenia [3]. For critically ill patients in the intensive care unit (ICU), the rapid loss of skeletal muscle mass (SMM) in the first few days of severe illness is an issue of great importance. This significant muscle breakdown, often referred to as “acute sarcopenia”, has a profound impact on the treatment outcome. Patients lose up to 15% of their total muscle mass in the first week after admission to the ICU due to immobility, systemic inflammation, and catabolic stress, which leads to prolonged ICU stays, delayed weaning from mechanical ventilation, and increased mortality [4,5].

Despite the increasing evidence on the negative effects of muscle wasting in critically ill patients, there is a lack of detailed understanding of how the acute loss of SMM during ICU treatment affects key clinical parameters, such as the duration of mechanical ventilation and the difficulty of weaning from ventilators. Understanding these effects may highlight the need for adapted therapeutic strategies, such as targeted nutritional interventions, early mobilization, and specialized physiotherapy, to decrease muscle loss and reduce complications related to sarcopenia. The EWGSOP report calls for increased consideration of muscle strength measurement to diagnose sarcopenia, but also emphasizes the importance of quantifying skeletal muscle mass or quality for a definite diagnosis [1]. Techniques such as magnetic resonance imaging (MRI) or computed tomography (CT), especially single-slice CT imaging of the spine, are recommended for accurate assessment of muscle mass in research settings [1]. However, most research focuses on determining the baseline muscle mass before critical illness, while the progression of the skeletal muscle index (SMI) during the ICU stay has been little studied. In particular, the analysis of routine CT images obtained during ICU treatment is rarely used, although these could provide valuable information on acute muscle loss.

In this retrospective analysis, we specifically examined the impact of SMM loss on ICU outcomes by quantifying the skeletal muscle cross-sectional area (CSA) in two single-slice CT images obtained at different time points during an ICU stay. We hypothesized that a significant decrease in SMM during the first two weeks of an ICU stay is associated with prolonged mechanical ventilation, regardless of the patients’ baseline SMM.

## 2. Materials and Methods

We analyzed patient records from our prospective database of the Department of Anesthesiology and Intensive Care at the Klinikum rechts der Isar, School of Medicine, Technical University of Munich, Germany, between 1 April 2017 and 30 April 2019 as described before [6,7]. This analysis is covered by the approval of the ethics committee of the School of Medicine and Health, Technical University of Munich (Ismaninger Straße 22, 81675 Munich, Chairperson Prof. Dr. G Schmidt, 518/16S) and was retrospectively registered in Clinical Trials NCT03666286 on 4 September 2018. The data of surgical critically ill patients were collected after obtaining written informed consent from the patients or their legal representative, in accordance with German law. Patients were analyzed if they received at least two CT scans, the first being administered −14 d to +0 d from ICU admission and the second being taken between +5 d to +21 d from ICU admission.

After identifying the patients who received two CT scans, we performed a two-step quality control process to ensure adequate image quality for measuring. First, we screened the images for exclusion criteria, which include (a) CT scans that do not include imaging of the vertebral levels Th10 to L5; (b) the presence of relevant artifacts; (c) CT scans that were not taken in the supine position; (d) CT scans constructed using a non-standard kernel (i.e., kernels used for other applications than soft tissue analysis); (e) a cropped field of view (the slice does not include all skeletal muscle of the desired region in axial view); and (f) pathologic changes that render the demarcation of the skeletal muscle impossible (for examples, see Supplementary Material: Figure S1). Second, we ensured a high image quality by assessing each image for the following three exclusion variables: (a) a large difference in rib area between the first and the second CT scan on the exact vertebral level; (b) degree of smaller streaking artifacts; and (c) difficulty differentiating skeletal muscle from the surrounding tissue (for example, the presence of substantial edema). If available, the L3 level was analyzed since its CSA has an ideal correlation with whole-body SMM [8]. If the L3 level was not part of the scan, we selected the best available vertebral level between Th10 and L5 to estimate whole-body SMM [9,10] Please see the Supplementary Material S1 for the distribution of vertebral levels. To ensure an identical position within the level between the images from consecutive scans, we analyzed slices showing the spinous process. Although a slice thickness of three millimeters was preferred, we prioritized images in which the skeletal muscle was easily identifiable, even if the slice thickness was higher or lower. If a certain image and, therefore, vertebral level was excluded in the quality insurance process, we moved to the next best one, as described by Derstine et al. [9]. We then performed the aforementioned quality control process again, until a usable slice was identified or the whole CT scan was excluded.

**Figure 1:**
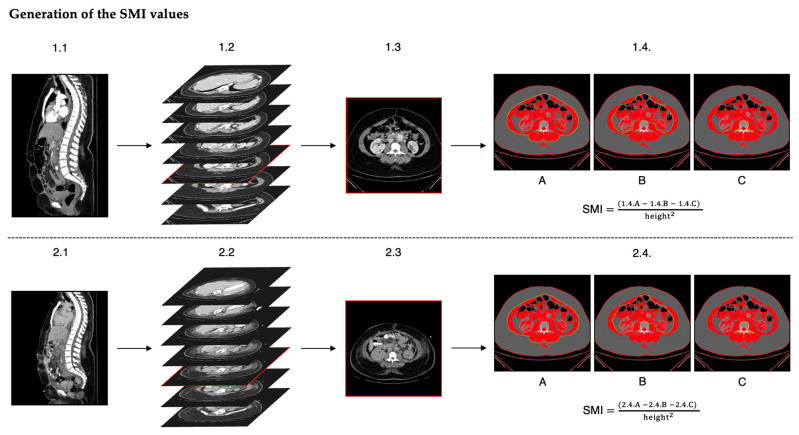
SMI value generation (workflow): After patients with two viable CT scans were identified, the best vertebral level of the first CT scan was chosen (1.1 & 1.2), and the two-step quality control process was performed (1.3). If the slice proved to be unusable, another level was chosen. If usable, the skeletal muscle cross sectional area was measured by delineating the outer skeletal muscle perimeter (A) the inner skeletal muscle perimeter (B) and the vertebra (C). With these measurements the SMI was calculated (1.4.A + B + C). This process was then repeated for the second CT scan (2.1 to 2.4.C).

We used the skeletal muscle index (SMI) [cm^2^/m^2^] as a surrogate for total body SMM. The SMI is calculated by dividing the CSA of the slice [cm^2^] by the squared height of the patient [m^2^] [11]. This normalizes the CSA, which inherently is closely correlated to the body size. To determine the CSA, we used the National Institutes of Health (NIH) ImageJ software (Version 1.53t), which represents a qualitatively equivalent alternative for body composition analyses as techniques using SliceOmatic (TomoVision, Montréal, QC, Canada) [12,13]. Following the steps, as previously described by Gomez-Perez et al. [14,15], we delineated the paraspinal muscles, abdominal muscles, and psoas muscles using a graphics tablet with a stylus pen (Wacom^®^ Intuos medium, Toyonodai, Kazo-shi, Saitama, Japan) to ensure comparability within the cohort. Within the delineated area, the CSA of the slice was calculated from the pixels that have Hounsfield unit values within the predefined and validated boundaries of −29 to +150 [16,17] (Figure 1). The observer measuring the SMM was blinded to the clinical outcomes of the patients.

The primary outcome was hours of mechanical ventilation. Secondary outcomes included the need for mechanical ventilation (yes/no), hours of mechanical ventilation of only the ventilated patients, need for tracheotomy (yes/no), ICU and hospital mortality, ICU and hospital length of stay, as well as the requirement (all yes/no) for extracorporeal membrane oxygenation (ECMO), dialysis, and resuscitation (defined as the necessity for defibrillation or mechanical compression of the thorax).

Data extracted from medical records and electronic patient data management systems included basic demographics and the respective department at ICU admission. Data collected after ICU admission included location before ICU admission, ICU admission category (sepsis, polytrauma, traumatic brain injury, non-traumatic brain injury, postoperative monitoring, cardiac failure, respiratory failure, and “other”), as well as standard laboratory and hemodynamic parameters. We used several scores to characterize the cohort: the Clinical Frailty Scale (CFS) to summarize the overall level of fitness or frailty [18,19], the baseline Glasgow Coma Scale (GCS) to assess each patient’s level of consciousness [20,21], the Acute Physiology And Chronic Health Evaluation II (APACHE-II) to assess the illness severity [22], the Sequential Organ Failure Assessment Score (SOFA) to assess the patient’s organ function [23], the Charlson Comorbidity Index (CCI) to predict the risk of one-year mortality [24,25], and the Barthel Index at hospital admission to systematically assess each patient’s ability to fulfill activities of daily living [26].

Statistical analysis was performed using R (Version 4.3.1) with R studio (1 September 2023+ 494, Foundation for Statistical Computing, Vienna, Austria). Discrete values are presented as absolute and relative numbers, and continuous variables are presented as medians with an interquartile range (IQR). A *p*-value of ≤0.05 was defined as statistically significant. The patients were divided into an atrophy group and a swelling group depending on whether the direction of absolute change in SMI was negative or positive, respectively. This direction of absolute change in SMI was calculated by subtracting the first SMI value from the second SMI value. Differences in clinical variables between the atrophy group and the swelling group were compared using Mann–Whitney U tests for continuous variables and Fisher’s exact tests for categorial variables. Three exploratory analyses were conducted. First, we analyzed the difference in mortality between the patients included in the final analysis and the rest of the recruited patients. This difference is depicted with a Kaplan–Meier diagram and tested with a log-rank test. Second, we examined the relationship between the first and second SMI value, which we represented using a scatter plot and quantified the linear relationship with a Pearson’s rho correlation. Third, we conducted univariate analysis to identify possible influencing factors for the direction of absolute change in SMI (defined as univariate *p*-value < 0.1). To adjust for possible confounders, logistical and linear regression models were analyzed using the results from the univariate analysis as well as APACHE II as a measurement for illness severity.

## 3. Results

### 3.1. Analyzed Patients

**Figure 2:**
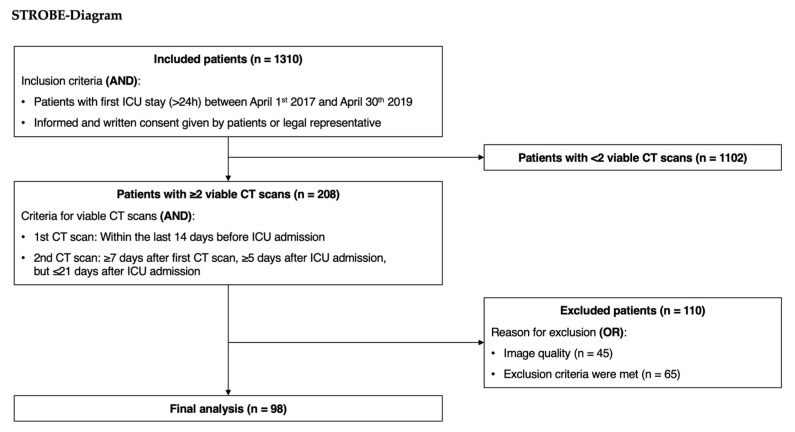
Diagram of included and excluded patients.

In total, 1310 patients from two surgical ICUs were included in the study. Out of these patients, 1102 participants did not receive at least two viable CT scans. Out of the remaining 208 patients, an additional 110 patients had to be excluded due to insufficient vertebral levels in the CT scan or poor image quality, leaving 98 participants for the final analysis (Figure 2).

### 3.2. Demographic Characteristics

In total, 70.4% (*n* = 69) of the patients were female. The median age was 68 (59,76) years, and the median body mass index was 25.0 (23.4, 28.0) kg/m^2^. Table 1 summarizes the patient characteristics.

### 3.3. SMI Characteristics

For each patient, two SMI values were computed, resulting in a total of 196 SMI values. The L3 level was used 63 times for the computation (See Supplementary Material: Table S1). In total, 38.8% (*n* = 38) of the patients fell into the swelling group (increase in SMI), while the other 61.2% (*n* = 60) were in the atrophy group (decrease in SMI). The median absolute change in SMI between the two measurements was −1.2 (−5.5, 2.3) cm^2^/m^2^. The median duration between the two CT scans was 14 (10,20) days, and the median SMI change per day was −0.12 (−0.33, 0.14) cm^2^/m^2^ (Table 2).

### 3.4. Outcomes

In univariate analyses, the atrophy group required significantly more hours of mechanical ventilation than the swelling group (415 vs. 42 h, *p* = 0.003) and had to be mechanically ventilated significantly more often (88.3% vs. 60.5%, *p* = 0.002; adj. OR 5.85 [1.90 to 20.40] *p* = 0.003). Comparing those patients with mechanical ventilation only, there was no significant difference in the duration of mechanical ventilation between the two groups (440 vs. 340 h, *p* = 0.3) (Table 3).

The swelling group had a significantly lower tracheotomy rate (50.0% vs. 23.7%, *p* = 0.011; adj. OR 3.96 [1.43 to 10.40] *p* = 0.009) and a significantly shorter duration of ICU stay (22 vs. 13 days, *p* = 0.045) in the univariate analyses. ICU mortality and hospital mortality showed no significant difference between the two groups (20.0% vs. 10.5%, *p* = 0.3 and 28.3% vs. 21.1%, *p* = 0.5, respectively). The need for ECMO, dialysis, and resuscitation also showed no significant difference when compared between the two groups (3 vs. 0, *p* = 0.3, 28 vs. 15, *p* = 0.5 and 12 vs. 2, *p* = 0.072, respectively) (Table 3). For all multivariate regression models, please see the Supplementary Material Tables S2–S5.

### 3.5. Exploratory Analysis

**Figure 3:**
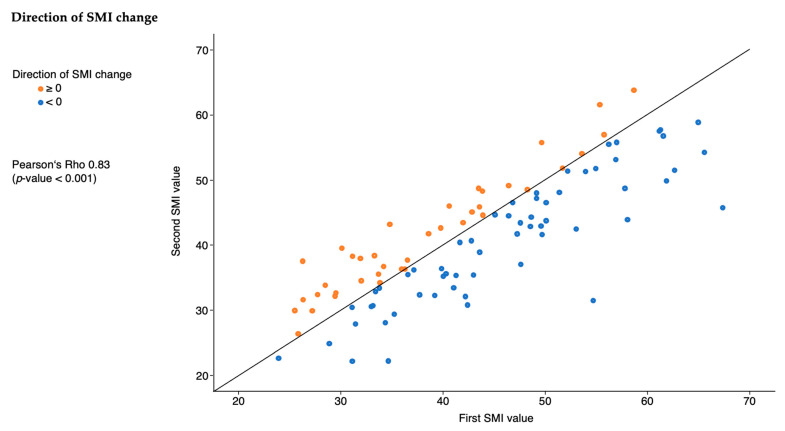
Direction of SMI change between the first and second measurement. Each dot represents one patient. Orange dots indicate that the second SMI value is larger than the first value (swelling). Blue dots indicate that the second SMI value is smaller than the first value (atrophy). The line indicates no change in SMI.

There was no significant difference in the direction of the SMI change depending on the magnitude of the SMI value generated form the first CT scan (Pearson’s rho: 0.83 (*p* < 0.001) (Figure 3).

**Figure 4:**
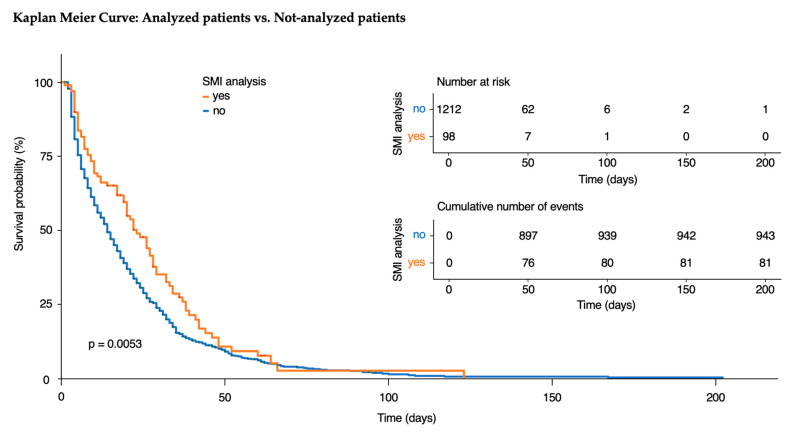
Survival probability of included and analyzed patients versus included but not analyzed patients presented using a Kaplan–Meier curve. The orange line indicates patients who were analyzed, and the blue line indicates patients who were not. Log rank test between the groups showed a significant difference in survival (*p* = 0.0053).

We found a significant difference in survival probability between the patients included in our final analysis compared to those who were included but not analyzed (*p* = 0.0053) (Figure 4).

Multivariate regression analysis indicated no significant association between a decrease in SMI and female sex (OR 2.04, 95% CI 0.81–5.20, *p* = 0.13), BMI categories (overweight OR 1.35, 95% CI 0.52–3.59, *p* = 0.54, obese OR 2.65, 95% CI 0.79–10.8, *p* = 0.14) and APACHE II score (OR 1.01,95% CI 0.95–1.08, *p* = 0.72) (See Supplementary Material: Table S6). These variables are measures of illness severity as well as factors that might influence the direction of absolute SMI change, as shown by our univariate analysis (See Supplementary Material: Table S7).

## 4. Discussion

This was one of the first studies to use two CT-derived measurements to detect change in SMI during an ICU stay as a parameter for predicting outcomes in critically ill surgical patients. A loss of SMM during the first weeks in the ICU was associated with prolonged mechanical ventilation. The increased likelihood of requiring mechanical ventilation and tracheotomy further supports this finding. Recent research has demonstrated a significant correlation between a low CT-measured SMM prior to critical illness and various outcomes, such as ICU admission in COVID-19 patients [27], successful extubation in critically ill COVID-19 patients [28], 90-day mortality in critically ill surgical patients [29], as well as 6-month mortality and disability at discharge [30].

Older studies also showed a statistically significant correlation between low SMM at ICU admission and the duration of mechanical ventilation and ICU length of stay [2]. Although these studies did not track changes in SMM over time, focusing instead on baseline measurements at ICU admission, they consistently demonstrated that low SMM adversely affects patient outcomes. However, our study diverged from these earlier analyses by focusing on the trajectory of SMI during the ICU stay. In 2019, Dusseaux et al. were among the first to evaluate changes in body composition during critical illness. In their pilot study, 25 ICU patients with sepsis were analyzed, and no statistically significant association was found between low SMM at ICU admission, alterations in SMM, and the duration of mechanical ventilation or ICU stay [31]. The discrepancy between their findings and ours may be explained by differences in the timing of CT scans (14 days vs. 10 days) and the larger surgical cohort in our study. Given that skeletal muscle loss correlates with the need for and duration of ventilation, our study highlights the need for preventive measures to halt or slow muscle loss, potentially reducing the risk of adverse ventilation-related outcomes. Nonetheless, when comparing the duration of mechanical ventilation between patients who experienced muscle swelling and those who experienced atrophy, no significant differences were found. Since diaphragm mass was not directly measured, no conclusions can be drawn about respiratory muscle strength, suggesting other factors contribute to the prolonged ventilation. Dusseaux et al. [31] also demonstrated that not only SMM quantity fluctuates, but also muscle density, which may reflect muscle function [31]. However, we did not assess this in our analysis.

Our study also showed that a decrease in SMM is a risk factor for prolonged ICU stays in univariate analysis. By comparing our results with ultrasound assessments (in most cases of the extremities), the loss of core muscles is found to be relatively milder than that of the extremities, where it is often reported as up to 15% [5]. In four studies where skeletal muscle was assessed at the level of the lumbar spine using CT scans, muscle loss varied. Lambell et al. found a loss of 15% of muscle mass during the first week in the ICU. Dusseaux et al. reported a loss of 4.29% over a period of 7 to 14 days [31]. Jung et al. reported a loss of 5.85% after 25 days, and a fourth study by Haines et al. compared CT scans before and after treatment and found changes of about 12% in the first 10 days [5]. However, it must be emphasized here that, even if the values were presented as relative changes, the studies are not focused on the SMI, but various entities, such as those associated with the psoas muscle or diaphragm, were calculated.

Given that decreased SMM impairs a patient’s ability to perform activities of daily living [32], extended ICU stays may be a consequence of the need for intensified nursing care. However, ICU or hospital mortality was not affected, potentially due to selection bias. Since only patients with two CT scans within a two-week period were included in this sub-analysis, it inherently represents a more favorable sub-cohort (survivor bias). Although the atrophy group exhibited a trend toward increased need for invasive therapies such as ECMO, dialysis, and resuscitation, further studies with larger patient cohorts are needed to confirm these findings. Future research should also explore whether these outcomes directly result from SMM atrophy, as we hypothesize for ECMO, dialysis, and resuscitation rates, or whether these treatments contribute to muscle atrophy. There was no correlation between changes in SMI and baseline SMI levels. Regardless of baseline SMI at ICU admission, critically ill patients either lost or gained SMM during their ICU stay.

As previously mentioned, a key difference between the study population and the general ICU population is the lower mortality rate observed in our cohort. This discrepancy may stem from the inclusion criterion of requiring two CT scans at least 7 days apart, which implies an ICU survival of at least 5 days. In actual results, the median difference even amounted to 14 days. Consequently, the study population may be less severely ill than the general ICU population.

### Limitations

As a limitation, only a small proportion of a large, heterogeneous ICU cohort could be included in the final analysis, making this an exploratory study. Although two CT scans were performed within the correct timeframes, 110 out of 208 patients (52.9%) had to be excluded due to issues such as image quality. However, we prioritized data quality for this first longitudinal study in critically ill patients, which led to the exclusion of 25% of patients. With the period of the first CT image of up to 14 days before admission to the intensive care unit, patients with a stable premorbid status may be confounded with patients who were already seriously ill, which has to be mentioned as limitation. On average, the first CT was performed one day before admission to the intensive care unit. This does not rule out bias, but it does allow a good assessment of the patient’s condition at the time of clinical deterioration and before mechanical ventilation.

Furthermore, CT scans expose patients to significant radiation, which presents its own risks [33], and the scans require additional resources for critically ill patients. This underscores the importance of developing alternative tools for assessing muscle mass. For this reason, we only evaluated routinely performed CTs, which, however, were not optimized for a precise visualization of the muscle tissue. The gold standard for muscle mass assessment is measurement at the level of the third lumbar vertebra (L3); however, in routine imaging, this region is not always captured with sufficient quality. Due to this limitation, we selected the most suitable alternative anatomical landmark for analysis, which may lead to bias. However, since only relative changes between measurements were analyzed, rather than absolute values, this potential bias was mitigated. Ultrasound measurement of the rectus femoris muscle at various time points during the ICU stay, as demonstrated by Fuest et al. [34] and Andrade-Junior et al. [35], may be a promising alternative. Since ultrasound can be performed bedside and does not emit radiation, it offers a safe and practical method for tracking changes in skeletal muscle mass and quality, as shown in a systematic review by Casey et al. [36]. Although ultrasonography is frequently used to assess muscle mass, particularly in studies, it has limitations such as operator dependence and the lack of clear, universally accepted thresholds. Above that, it does not allow for direct assessment of core muscles. In contrast, CT scans provide objective, reproducible data. The evaluation of routine CT scans performed for other clinical indications could provide a valuable additional tool to assess muscle changes without the need for additional examinations. We believe that the use of routine CT scans can add significant value in the detection and assessment of muscle changes as the data are accurate and reproducible.

Another limitation that has to be mentioned is the high proportion of older patients with a subsequently increased likelihood of sarcopenia, which could lead to bias. However, it must be stated here that we are focusing on the percentage change in muscle mass per day per patient, regardless of baseline values. Furthermore, we were able to show that the direction of index development was independent of baseline values. Furthermore, it should be noted that some of the patients had already been hospitalized for a longer period of time before being admitted to the intensive care unit. However, the main objective of our study is the relative change per day after ICU admission and its effect on outcome. This is independent of baseline values, as we have shown in the results.

Finally, the pathophysiological mechanisms behind “muscle swelling” in some patients remain unclear. Our study did not include measurements of muscle density or tissue composition, so further research is needed to understand this phenomenon.

## 5. Conclusions

This study suggests that a decrease in skeletal muscle index (calculated with a single slice extracted from a CT scan at two different timepoints) in critically ill patients within the first two weeks is negatively associated with ventilation parameters, such as the necessity and duration of mechanical ventilation and tracheotomy rates.

## Figures and Tables

**Table 1:** Demographic characteristics.

	*n* = 98
Female gender	69 (70.4%)
BMI (kg/m^2^)	25.0 (23.4,28.0)
Underweight	1 (1.0%)
Normal	48 (49.0%)
Overweight	32 (32.7%)
Obese	17 (17.3%)
Age (years)	68 (59,76)
≤50	14 (14.3%)
51–65	30 (30.6%)
66–80	48 (49.0%)
>80	6 (6.1%)
Department	
Neurosurgery	14 (14.3%)
Thoracic Surgery	1 (1.0%)
Abdominal Surgery	55 (56.1%)
Vascular Surgery	8 (8.2%)
Trauma Surgery	3 (3.1%)
Neurology	5 (5.1%)
Enterology	3 (3.1%)
Gynecology	1 (1.0%)
Urology	2 (2.0%)
Internal Medicine	4 (4.1%)
Other	2 (2.0%)
Admission	
From home	67 (68.4%)
From hospital	30 (30.6%)
From nursing home	1 (1.0%)
ICU admission category	
Sepsis	20 (20.4%)
Polytrauma	5 (5.1%)
Traumatic brain injury	2 (2.0%)
Non traumatic brain injury	4 (4.1%)
Postoperative monitoring	27 (27.6%)
Cardiac failure	8 (8.2%)
Respiratory failure	35 (35.7%)
Other	22 (22.4%)
Frail (CFS 5–9)	14 (14.3%)
GCS	15 (11,15)
APACHE II	14 (10,19)
SOFA	7 (5,9)
CCI	2 (0,3)
Barthel Score at hospital admission	30.0 (30.0, 30.0)

Data are expressed as median [interquartile range] or counts (percentages). BMI: body mass index, CFS: Clinical Frailty Scale, GCS: Glasgow Coma Scale, APACHE II: Physiology And Chronic Health Evaluation II, SOFA: Sequential Organ Failure Assessment Score, CCI: Charlson Comorbidity Index.

**Table 2:** SMI characteristics.

	*n* = 98
Days between CT scans (days)	14 (10,20)
<0	16 (12,20)
≥0	12 (9,18)
Direction of change in SMI	
<0	60 (61.2%)
≥0	38 (38.8%)
Absolute change in SMI (cm^2^/m^2^)	−1.2 (−5.5, 2.3)
Percentage change in SMI (%)	−3 (−12, 5)
Change in SMI per day ((cm^2^/m^2^)/days)	−0.12 (−0.33, 0.14)

Data are expressed as median [interquartile range] or counts (percentages). Direction of change in SMI: <0 = second SMI < first SMI and ≥0 = second SMI ≥ first SMI, Absolute change in SMI: second SMI − first SMI, Percentage change in SMI: (second SMI − first SMI) divided by first SMI, Change in SMI per day: (second SMI − first SMI) divided by days between the CT scans.

**Table 3:** Primary and secondary outcomes.

Variables	Atrophy (*n* = 60)	Swelling (*n* = 38)	Univariate *p* Value	Multivariate	
				Estimate/OR [95% CI]	*p* Value
Hours of ventilation	415 (116,594)	42 (0, 491)	0.003	155 (−17 to 328) ^a^	0.076
Ventilated during ICU stay	53 (88.3%)	23 (60.5%)	0.002	5.85 (1.90 to 20.40) ^b^	0.002
If ventilated, hours of ventilation	440 (186, 668)	340 (72, 673)	0.3		
Tracheotomy	30 (50.0%)	9 (23.7%)	0.011	3.96 (1.43 to 10.40) ^b^	0.006
ICU mortality	12 (20.0%)	4 (10.5%)	0.3		
Hospital mortality	17 (28.3%)	8 (21.1%)	0.5		
ICU length of stay (days)	22 (14,35)	13 (5,28)	0.045	5.2 (−3.0 to 13.0) ^a^	0.2
Hospital length of stay (days)	48 (31,61)	44 (31,64)	0.8		
ECMO	3 (5.0%)	0 (0.0%)	0.3		
Dialysis	28 (46.7%)	15 (39.5%)	0.5		
Resuscitation	12 (20.0%)	2 (5.3%)	0.072		

Data are expressed as median [interquartile range] or counts (percentages). All multivariate models are corrected for gender, BMI, and Apache score. CI: confidence interval, OR: odds ratio, ^a^: estimate of the linear regression model, ^b^: OR of the binary logistic regression model.

## Data Availability

The original contributions presented in this study are included in the article. Further inquiries can be directed corresponding author.

## Supplementary Materials

The following supporting information can be downloaded at: https://www.mdpi.com/article/10.3390/jcm13247772/s1, Table S1: Percentages of vertebral levels; Table S2: Multivariate regression models—ventilated during ICU stay, Table S3: Multivariate regression models—hours of ventilation, Table S4: Multivariate regression models—tracheotomy, Table S5: Multivariate regression models—ICU length of stay, Table S6: Logistical regression analysis, Table S7: Univariate analysis, Figure S1: Exclusion criteria: (**A**) CT scans that do not pass through the vertebral levels Th10 to L5; (**B**) the presence of artifacts (for example streak artifacts due to metal); (**C**) CT scans that were not taken in the supine position; (**D**) CT scans that did not include a soft-tissue window series (for example bone window); (**E**) a cut-off border of the skeletal muscle; (**F**) pathologic changes which render the demarcation of the skeletal muscle impossible (for example abscess in the skeletal muscle: highlighted with the red arrow).
