# Supplementary material for: Skeletal Muscle Mass Loss Leads to Prolonged Mechanical Ventilation and Higher Tracheotomy Rates in Critically Ill Patients
Source: J Clin Med. 2024 Dec 19;13(24):7772. doi: 10.3390/jcm13247772 (PMC11728401; doi:10.3390/jcm13247772)
Supplement: Supplementary file 1 [file jcm-13-07772-s001.zip › jcm-3353200-supplementary.pdf]

Supplementary Material

Table S1. Percentages of vertebral levels.

Vertebral Level	Number of Participants (n = 98)
Th10	0 (0.00%)
Th11	6 (6.12%)
Th12	5 (5.10%)
L1	11 (11,22%)
L2	4 (4,08%)
L3	63 (64,28%)
L4	8 (8,16%)
L5	1 (1,02%)

This table shows the number of times each vertebral level was used to generate the SMI value for a participant. Data are expressed in counts (percentages).

Table S2. Multivariate regression models—ventilated during ICU stay.

	OR	95% CI	*p* Value
Change in SMI per day	5.85	1.90, 20.4	0.003
Female gender	1.01	0.30, 3.21	>0.9
BMI (categories)			0.29
Normal			
Obese	0.82	0.18, 4.15	0.8
Overweight	2.54	0.68, 10.7	0.2
APACHE II	1.19	1.07, 1.37	0.005

Logistical regression model for ventilation during ICU stay with the factors SMI change, female gender, body mass index (BMI), and APACHE-II Score. These variables are measures of illness severity as well as factors that might influence the direction of absolute SMI change, as shown by our univariate analysis (Table S1). OR: odds ratio, CI: confidence interval, BMI: Body mass index, APACHE II: Physiology And Chronic Health Evaluation II.

Table S3. Multivariate regression models—hours of ventilation.

	OR	95% CI	*p* Value
Change in SMI per day	56	−161, 272	0.6
Female gender	−13	−231, 205	>0.9
BMI (categories)			0.6
Normal			
Obese	−120	−392, 151	0.4
Overweight	−79	−302, 143	0.5
APACHE II	2.6	−11, 17	0.7

Logistical regression model for hours of ventilation with the factors SMI change, female gender, body mass index (BMI), and APACHE II score. These variables are measures of illness severity as well as factors that might influence

the direction of absolute SMI change, as shown by our univariate analysis (Table S1). OR: odds ratio, CI: confidence interval, BMI: body mass index, APACHE II: Physiology And Chronic Health Evaluation II.

Table S4. Multivariate regression models—tracheotomy.

	OR	95% CI	*p* Value
Change in SMI per day	3.69	1.43, 10.4	0.006
Female gender	0.52	0.19, 1.37	0.2
BMI (categories)			0.8
Normal			
Obese	0.94	0.28, 3.13	>0.9
Overweight	1.27	0.46, 3.57	0.6
Underweight			>0.9
APACHE II	1.07	1.00, 1.15	0.039

Logistical regression model for tracheotomy with the factors SMI change, female gender, body mass index (BMI), and APACHE II score. These variables are measures of illness severity as well as factors that might influence the direction of absolute SMI change, as shown by our univariate analysis (Table S1). OR: odds ratio, CI: confidence interval, BMI: body mass index, APACHE II: Physiology And Chronic Health Evaluation II.

Table S5. Multivariate regression models—ICU length of stay.

	OR	95% CI	*p* Value
Change in SMI per day	5.2	−3.0, 13	0.2
Female gender	−1.5	−10, 7.2	0.7
BMI (categories)			0.7
Normal			
Obese	−3.9	−15, 7.1	0.5
Overweight	1.6	−7.4, 11	0.7
Underweight	17	−23, 56	0.4
APACHE II	0.23	−0.35, 0.81	0.4

Logistical regression model for ICU length of stay with the factors SMI change, female gender, body mass index (BMI), and APACHE II score. These variables are measures of illness severity as well as factors that might influence the direction of absolute SMI change, as shown by our univariate analysis (Table S1). OR: odds ratio, CI: confidence interval, BMI: body mass index, APACHE II: Physiology And Chronic Health Evaluation II.

Table S6. Logistical regression analysis.

	OR	95% CI	*p* Value
Female gender	2.04	0.81, 5.20	0.13
BMI (categories)			0.29
Normal			
Obese	2.65	0.79, 10.8	0.14
Overweight	1.35	0.52, 3.59	0.54
APACHE II	1.01	0.95, 1.08	0.72

Logistical regression model with the factors female gender, body mass index (BMI), and APACHE II Score. These variables are measures of illness severity as well as factors that might influence the direction of absolute SMI change, as shown by our univariate analysis (Table S1). OR: odds ratio, CI: confidence interval, BMI: body mass index, APACHE II: Physiology And Chronic Health Evaluation II.

Table S7. Univariate analysis.

Factors	Atrophy Group (n = 60)	Swelling Group (n = 38)	P Value
Female gender	46 (76.7%)	23 (60.5%)	0.088
BMI (kg/m^2)	25.6 [23.7, 29.2]	24.6 [21.6, 26.5]	0.021
BMI (categories)			0.3
Underweight	0 (0.0%)	1 (2.6%)	
Normal	27 (45.0%)	21 (55.3%)	
Overweight	20 (33.3%)	12 (31.6%)	
Obese	13 (21.7%)	4 (10.5%)	
Age (years)	66 [57, 75]	69 [60, 77]	0.3
Age (categories)			0.5
≤50	11 (18.3%)	3 (7.9%)	
51–65	17 (28.3%)	13 (34.2%)	
66–80	29 (48.3%)	19 (50.0%)	
>80	3 (5.0%)	3 (7.9%)	
Department			0.8
Neurosurgery	7 (11.7%)	7 (18.4%)	
Thoracic Surgery	0 (0.0%)	1 (2.6%)	
Abdominal Surgery	33 (55.0%)	22 (57.9%)	
Vascular Surgery	4 (6.7%)	4 (10.5%)	
Trauma Surgery	2 (3.3%)	1 (2.6%)	
Neurology	4 (6.7%)	1 (2.6%)	
Enterology	3 (5.0%)	0 (0.0%)	
Gynecology	1 (1.7%)	0 (0.0%)	
Urology	2 (3.3%)	0 (0.0%)	
Internal Medicine	3 (5.0%)	1 (2.6%)	
Other	1 (1.7%)	1 (2.6%)	
Admission			0.9
From home	40 (66.7%)	27 (71.1%)	
From hospital	19 (31.7%)	11 (28.9%)	
From nursing home	1 (1.7%)	0 (0.0%)	
Ventilated during ICU stay	39 (65.0%)	21 (55.3%)	0.3
ICU admission category			
Sepsis	14 (23.3%)	6 (15.8%)	0.4
Polytrauma	5 (8.3%)	0 (0.0%)	0.2
Traumatic brain injury	1 (1.7%)	1 (2.6%)	>0.9
Non traumatic brain injury	3 (5.0%)	1 (2.6%)	>0.9
Postoperative monitoring	14 (23.3%)	13 (34.2%)	0.2
Cardiac failure	7 (11.7%)	1 (2.6%)	0.15
Pulmonary failure	24 (40.0%)	11 (28.9%)	0.3
Other	10 (16.7%)	12 (31.6%)	0.085
Frail (CFS 5–9)	9 (15.0%)	5 (13.2%)	0.8
GCS	15 [12, 15]	15 [9, 15]	0.8

APACHE 2	14 [11, 18]	14 [10, 19]	0.6
SOFA	7 [5, 9]	7 [4, 9]	0.7
CCI	2 [0, 3]	2 [0, 4]	0.2
Barthel Score [1]	30.0 [30.0, 30.0]	30.0 [30.0, 30.0]	0.6
Barthel Score [1]			0.3
10	1 (1.7%)	0 (0.0%)	
15	1 (1.7%)	1 (2.6%)	
20	1 (1.7%)	4 (10.5%)	
25	6 (10.0%)	2 (5.3%)	
30	51 (85.0%)	31 (81.6%)	

Data are expressed as median [interquartile range] or counts (percentages). BMI: body mass index, CFS: Clinical Frailty Scale, GCS: Glasgow Coma Scale, APACHE II: Physiology And Chronic Health Evaluation II, SOFA: Sequential Organ Failure Assessment Score, CCI: Charlson Comorbidity Index. [1] at hospital admission.

Quality Control Process: Exclusion Criteria

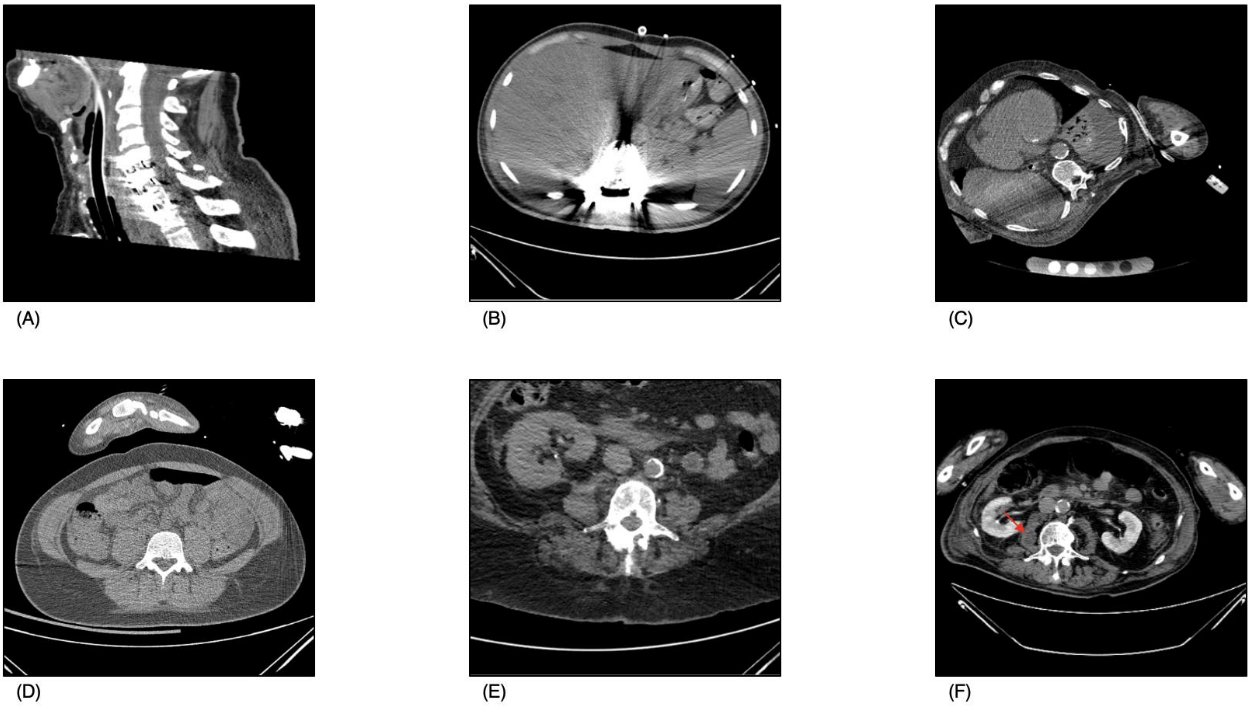

Figure S1. Exclusion criteria: (**A**) CT scans that do not pass through the vertebral levels Th10 to L5; (**B**) the presence of artifacts (for example streak artifacts due to metal); (**C**) CT scans that were not taken in the supine position; (**D**) CT scans that did not include a soft-tissue window series (for example bone window); (**E**) a cut-off border of the skeletal muscle; (**F**) pathologic changes which render the demarcation of the skeletal muscle impossible (for example abscess in the skeletal muscle: highlighted with the red arrow).
